# Flexible Ultrasonic Transducer Array with Bulk PZT for Adjuvant Treatment of Bone Injury †

**DOI:** 10.3390/s20010086

**Published:** 2019-12-22

**Authors:** Huicong Liu, Jiangjun Geng, Qifeng Zhu, Lue Zhang, Fengxia Wang, Tao Chen, Lining Sun

**Affiliations:** School of Mechanical and Electric Engineering, Jiangsu Provincial Key Laboratory of Advanced Robotics, Soochow University, Suzhou 215123, China; hcliu078@suda.edu.cn (H.L.); 20185229001@stu.suda.edu.cn (J.G.); qifengz@outlook.com (Q.Z.); chent@suda.edu.cn (T.C.); lnsun@hit.edu.cn (L.S.)

**Keywords:** piezoelectric, micromachined, flexible substrate, ultrasonic transducer array

## Abstract

Flexible electronic devices are developing rapidly, especially in medical applications. This paper reports an arrayed flexible piezoelectric micromachined ultrasonic transducer (FPMUT) with a sandwich structure for adjuvant treatment of bone injury. To make the device conformable and stretchable for attaching to the skin surface, the flexible substrate of polydimethylsiloxane (PDMS) was combined with the flexible metal line interconnection between the bulk lead zirconate titanate (PZT) arrays. Simulations and experiments were carried out to verify the resonant frequency and tensile property of the reported FPMUT device. The device had a resonant frequency of 321.15 KHz and a maximum sound pressure level (SPL) of 180.19 dB at the distance of 5 cm in water. In addition, detailed experiments were carried out to test its acoustic performance with different pork tissues, and the results indicated good ultrasound penetration. These findings confirm that the FPMUT shows unique advantages for adjuvant treatment of bone injury.

## 1. Introduction

Ultrasound is widely used in the field of medicine, especially in elderly rehabilitation [1,2,3], because of its excellent mechanical effects. This includes kidney stone lithotripsy with ultrasonic diagnostic technology and noninvasive tumor therapy with high-intensity focused ultrasound (HIFU) [4,5,6,7,8,9,10]. Recent research on the effect of ultrasound on chondrocyte proliferation and matrix production of human articular cartilage has shown the potential of low-intensity pulsed ultrasound (LIPU) for adjuvant treatment of bone injury [11,12]. As the core component of an ultrasonic system, acoustic transducers are mostly based on piezoelectric or capacitive mechanisms. Piezoelectric ultrasonic transducers convert electrical signals into mechanical energy by means of the piezoelectric effect of materials and then transmit them. A capacitive ultrasonic transducer is a kind of electrostatic transducer. Its diaphragm generates ultrasonic sound by virtue of electrostatic attraction. However, for traditional ultrasound transducers, the two-dimensional arrays with rigid substrates are incapable of adapting to the human body curve, while the line arrays are difficult to operate [13,14]. Thus, the reported ultrasound transducers are both uncomfortable and inconvenient for long-term adjuvant treatment of bone injury. 

With advancements in the material and fabrication process of flexible electronic devices [15,16,17,18,19,20,21,22,23,24,25,26,27,28], flexible ultrasound wearable technology has been greatly improved. Most of the current flexible ultrasonic transducers integrate micro piezo elements on a flexible substrate to make the whole device flexible. Mastronardi et al. [29] proposed a promising wearable ultrasound technology based on a piezoelectric transducer realized on flexible, highly oriented aluminum nitride with significant mechanical displacement in spite of being attached on a rigid support. Lee et al. [30] proposed a flexible piezoelectric micromachined ultrasonic transducer (FPMUT) that could be used to study brain stimulation by ultrasound. The ultrasound transducer array was then strongly bonded onto a polydimethylsiloxane (PDMS) substrate to achieve flexibility. By measuring the ultrasound output pressure, the PMUT showed a sound intensity (Isppa) of 44 mW/cm^2^ at 80 V, which is high enough for low-intensity ultrasound brain stimulation. A flexible ultrasonic device using 4 × 4 arrayed bulk lead zirconate titanate (PZT) with a high resonant frequency of 2 MHz has been reported [31,32]. Experiments showed the frequency difference of each element was within 3%, and the whole device could be well fitted to a cylindrical surface with a radius of 1 cm. Despite great advancements in PMUT, traditional ultrasonic treatment equipment cannot be closely fitted with the human skin, and its lack of comfortability and portability still limits its application in the medical field. To the best of our knowledge, there have been few reports on flexible ultrasonic transducers for ultrasonic-assisted treatment of bone injury due to the limitations of the device structure. 

In this work, a FPMUT array with a sandwich structure was constructed by combining rigid piezoelectric ceramics with a flexible substrate and a flexible electrode. The FPMUT showed excellent flexibility and could fit the human skin and tissues. Simulations and experiments were carried out to verify the resonant frequency and tensile property of the reported FPMUT device. Results showed the FPMUT could be stretched by 25%, which meets the needs of biological deformation. The FPMUT could achieve a resonant frequency of 321.15 KHz and a maximum sound pressure level (SPL) of 180.19 dB at the distance of 5 cm in water, which is comparable to the reported results. In addition, different pork tissues were used to demonstrate its potential application in the adjuvant treatment of bone injury. The flexible structure and excellent performance make the FPMUT a good candidate as a wearable ultrasound device. 

## 2. Experiment

### 2.1. Design of the FPMUT Array

**Figure 1:**
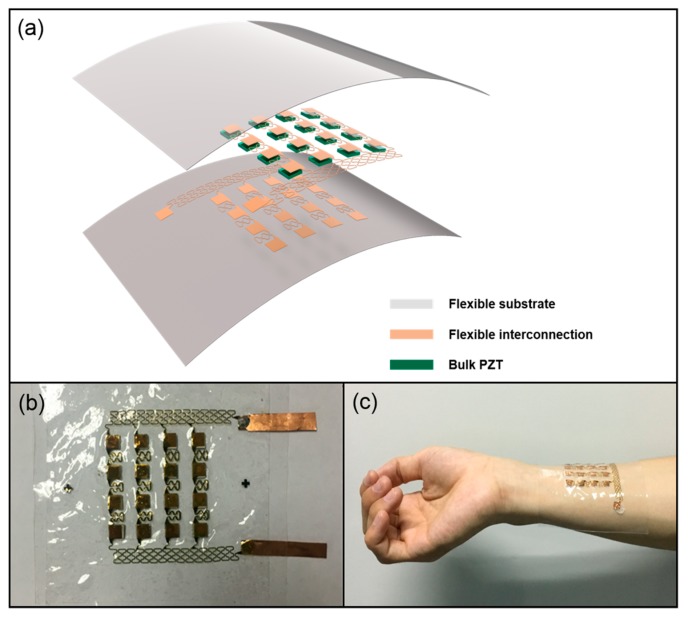
(**a**) Schematic drawing of the flexible piezoelectric micromachined ultrasonic transducer (FPMUT) array. (**b**) Photograph of the FPMUT array. (**c**) The FPMUT attached to skin of the arm.

A schematic diagram of the proposed FPMUT array is shown in Figure 1a. The device consists of a bulk PZT array of 4 × 4 units sandwiched by interconnection and PDMS films. Taking into consideration the convenience of actual processing and procurement, the bulk PZT has a thickness of 1 mm, and the typical PZT element is 5 mm × 5 mm × 1 mm (purchased from Baoding Shengke co. LTD). This is relatively common and makes it easy for use in later processes. A 0.01 mm silver electrode is required on both sides of the PZT. The 100 µm thick top and bottom substrates are made of flexible PDMS films due to their low Young’s modulus and high dielectric constant. Flexible polyimide (PI) are used as the protective layer on both the top and bottom sides of the electrode. The thickness of each PI layer is about 2.4 µm, and the Cu electrodes are patterned on the PI layers, which is combined with the PZT array by a low-temperature solder paste. The interconnection is stretched through the flexibility of PI materials and the tensile properties of the structural design. The combination of flexible interconnections, the PZT array, and flexible substrates form the FPMUT array. 

When alternating current is passed through the flexible interconnection with the piezoelectric ceramic array, the piezoelectric ceramic will deform due to the piezoelectric effect. At the same time, the frequency of the generated ultrasound is related to the frequency of the input voltage. Figure 1b shows a picture of the sample device, and Figure 1c shows a demonstration of the device being attached to skin.

### 2.2. Fabrication Process of the FPMUT Array

**Figure 2:**
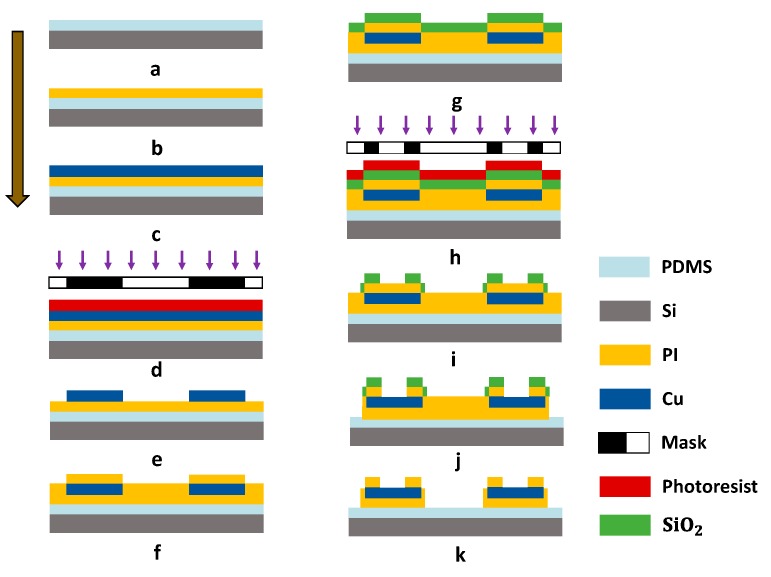
Process flow of the FPMUT.

The fabrication process of the FPMUT is shown in Figure 2. First, a 4 inch silicon (Si) wafer consisting of about 100 µm PDMS layer was used for the temporary substrate (mixed at 10:1 ratio, 3000 rpm for 30 s, at 80 °C for 3 h). Then, the PDMS temporary substrate was exposed to oxygen plasma to enhance the vitality of the surface (for 60 s). A 2.4 µm thick layer of PI was used as the protective layer (2000 rpm for 60 s, at 150 °C for 4 min and at 210 °C for 1 h). Then, a 400 nm thick layer of copper (Cu) was deposited by electron beam evaporation onto the PI (type of Ei-5z, at 200 °C, 5 × 10^−5^ Pa, for evaporation rate of 1 Å/s). A 1.5 μm thick AZ5214 positive photo resist (PPR) was spin-coated on the Cu layer. This was followed by a UV photolithography and developing process to obtain the patterned PPR. The Cu layer was patterned as serpentine structures through photolithography and etching (${\mathrm{CH}}_{3}$COOH/$H_{2}O_{2}/H_{2}O$ = 1:2:10). A second, 2.4 μm thick layer of PI and a 150 nm silicon oxide (${\mathrm{SiO}}_{2}$) covered the entire structure. Next, photolithography (AZ5214 PPR), reactive ion etching (RIE), and oxygen plasma etching patterned the layers of PI in a geometry matched to the metal traces (20 Sccm $O_{2}$, 80 mT, 200 W for 55 min). The residue ${\mathrm{SiO}}_{2}$ mask was removed using buffered oxide etchant (BOE 1:20). Finally, the flexible interconnection was retrieved using water-soluble tape for aligned transfer to the device substrate. The entire device with flexible interconnection was put in water for a few minutes, and the tape was gradually dissolved. PZT elements and flexible interconnections were bonded through the use of low-temperature solder paste (${\mathrm{Sn}}_{42}{\mathrm{Bi}}_{58}$). A clean squareglass was placed in the container with some trimethylchlorosilane (TMCS) as releasing agents. The container was sealed with preservative film for one night for volatilization of the mold release agents. After surface treatment on the square glass with mold releasing agents, a 200 μm thick PDMS layer was spin-coated onto the square glass. The PDMS film, as a flexible substrate, could been peeled off completely after a curing process at 80 °C for 3 h. We used this layer as the bottom layer of the device. Both top and bottom layers were fabricated using the method mentioned above, and another surface of the PZT unit was then bonded with the top layers using the low-temperature solder paste. The top and bottom layers with PDMS were pasted by fresh PDMS solution to form the complete device.

**Figure 3:**
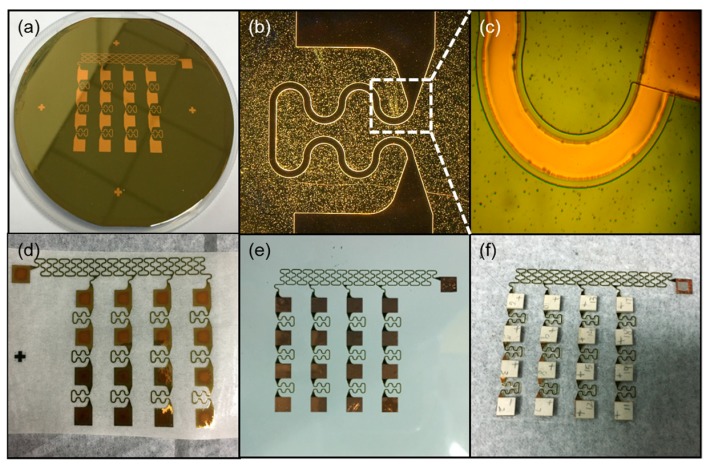
Effects of flexible interconnection in different steps. (**a**) The second layer of polyimide (PI) for the protective layer. (**b**) The Cu layer was patterned as serpentine structures. (**c**) The ${\mathrm{SiO}}_{2}$ hard mask after photolithography. (**d**) The flexible interconnection was retrieved by water-soluble tape. (**e**) The flexible interconnection was transferred on a new PDMS layer. (**f**) The flexible interconnection and the lead zirconate titanate (PZT) array were combined.

The effect of flexible interconnection in different steps is shown in Figure 3, where the shape of the electrode and protective layer can be seen. Figure 3a shows the effect of the second layer of PI covered by the whole device, while Figure 3b shows the Cu layer that was patterned as serpentine structures. Due to the maximum stress of the connecting part, the shape was used to ensure that the interconnection was not broken while stretching. We can see from Figure 3c that the ${\mathrm{SiO}}_{2}$ hard mask after photolithography was a little wider than the copper layer. The purpose of this step was to allow the PI layer to wrap the copper layer well for the future. Figure 3d–f shows the encapsulation process of the device. Figure 3d shows that the flexible interconnection was retrieved by water-soluble tape. This is a very simple method to transfer flexible interconnection. Figure 3e shows the interconnection being transferred on another new PDMS layer. We combined the flexible interconnection with PZT array using low-temperature solder paste, as shown in Figure 3f.

## 3. Results and Discussion 

### 3.1. Simulation of the FPMUT

#### 3.1.1. Mechanical Tensile Properties of the Flexible Interconnection

**Figure 4:**
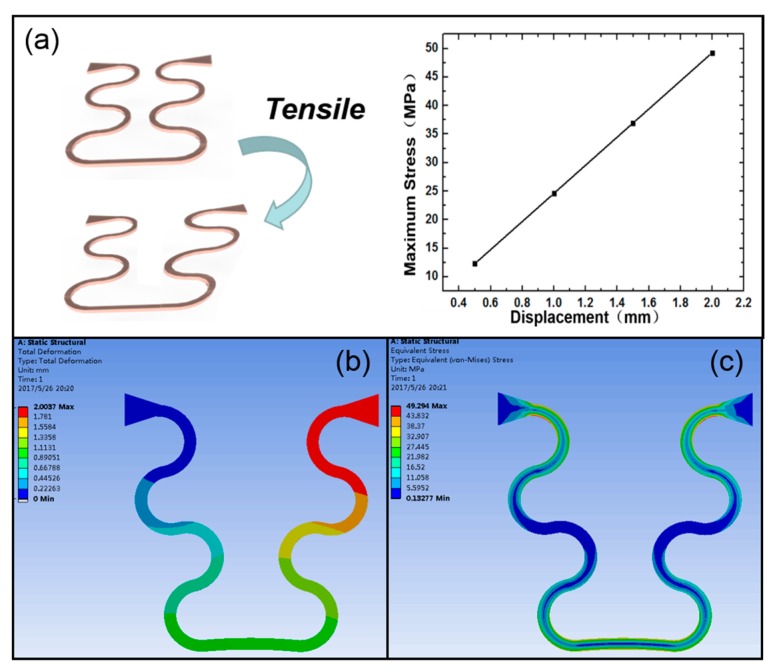
Simulation of mechanical tensile properties of the flexible interconnection. (**a**) The relationship between the stress and displacement. (**b**) The change in the shape of the flexible interconnection. (**c**) The distribution of internal stresses in the interconnection.

Figure 4 shows the tensile property of the curved part in the flexible interconnection, while Table 1 gives parameters of the material that was used in the simulation. The copper layer was too thin to account for the overall structure. Figure 4a shows that the maximum stress was linear with tensile displacement. As can be seen in Figure 4b–c, the change in the shape of the flexible interconnection and the distribution of strain were distinctively reflected. In the simulation, one end of the flexible electrode was fixed, and the other end was displaced. The total width of the flexible electrode between the two piezoelectric ceramics was 3.25 mm, and the maximum displacement set in the simulation was 2 mm. Therefore, the tensile strain could reach 61.5%, which is much higher than the maximum tensile strain of human skin (20%). In this case, the largest strength of the flexible interconnection was up to 49 MPa, and the maximum tensile strength of PI was 150 MPa. These results show that the tensile property is adequate to fit the whole device for contact with skin surfaces.

#### 3.1.2. Resonant Frequency of the Piezoelectric Element

To study the transmission sensitivity, a finite element model (FEM) of a single piezoelectric ceramic block based on the variation principle and subdivision interpolation was built, where the analysis of piezoelectric effects with structural mechanics models was employed. To solve piezoelectric elastic vibration problems, we used the Hamilton variation principle. Here, the structural vibration analysis of the entire continuum is divided into finite elements. These units are continuous at the boundary, and the parameters at the boundary nodes are regarded as unknown quantities. The method of constructing interpolation function can be given an unknown node relationship value and unit of arbitrary values so as to establish equations that satisfy the continuum approximation. Piezoelectric ceramics are anisotropic materials. The mechanical boundary and the electrical boundary condition determine their four piezoelectric equations, and the direction of polarization determines the transformation of their characteristic parameter matrix. For the structure of the piezoelectric transducer, the *e* type piezoelectric equation with a mechanical short circuit can be expressed as follows:

(1) $$\left\{\begin{array}{c} T=c^{E}S-e_{t}E\\ D=eS+\varepsilon^{s}E \end{array}\right\}$$

where T is the stress vector, D is the electric displacement vector, $c^{E}$ is the elastic coefficient matrix, S is the strain vector, E is the electric field vector, e is piezoelectric stress constant matrix, $e_{t}$ is the transposed matrix of e, and $\varepsilon^{s}$ is the dielectric constant matrix.

After the hybrid meshes and the variational processing method, the following equation can be obtained:

(2) $$\left(K-\omega^{2}M\right)U=PV$$

(3) $$P_{t}U+C_{0}V=Q$$

where K is the total stiffness matrix, P is the total electromechanical coupling vector, $P_{t}$ is the transposed matrix of P, M is the total mass matrix, U is the displacement vector of the piezoelectric node, ω is the angular frequency of simple harmonic vibration, $C_{0}$ is the clamping capacitance, V is the voltage between electrodes, and Q is the amount of charge on the electric pole. The characteristic parameters of the piezoelectric model can be calculated using Equations (2) and (3).

**Figure 5:**
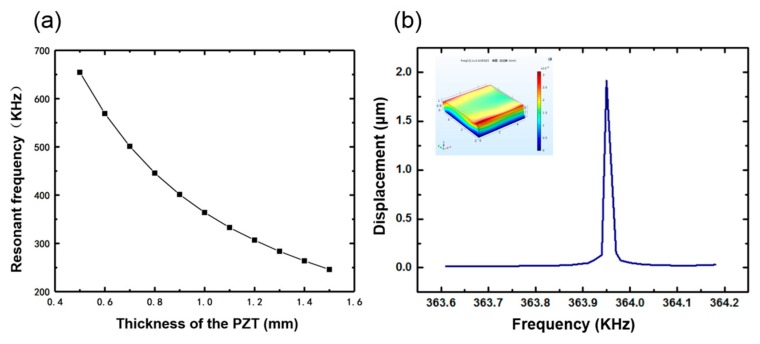
(**a**) Resonant frequency of the single element with different thicknesses. (**b**) The relationship between frequency and displacement.

While the PMDS layer and the flexible interconnection is not taken into consideration, the 100 μm low-temperature solder paste layer is defined, so it is consistent with the actual situation. According to the structure of the device, the whole model should be fixed to the bottom surface. The single piezoelectric ceramic block is defined as a piezoelectric material in the modeling. No initial stress is considered in the model; hence, the simulated results are taken as the ideal values. Piezoelectric ceramic blocks with different thicknesses have a certain resonance frequency, which can be seen in Figure 5a. Considering the convenience of processing and procurement, the size of the piezoelectric ceramic selected in this design was 5 mm × 5 mm × 1 mm. Figure 5b shows the deflection amplitude of the average displacement of the plane against frequency. At 10 V voltage, the simulated resonance of the single element was 363.95 KHz, and the average surface displacement was about 1.915 μm. The small picture in the upper left corner is the vibration mode of the device at the resonant frequency.

#### 3.1.3. Sound Field Distribution in Water

The sound intensity of a single piezoelectric ceramic block can be calculated by Equations (4) and (5).

(4) $$I=\frac{\rho c\omega_{0}^{2}A^{2}}{2}$$

(5) $$\omega_{0}=2\pi f$$

where I is the sound intensity, ρ is the medium density, c is the speed of sound propagation in the medium, A is the amplitude, $\omega_{0}$ is the angular velocity, and f is the frequency. The sound intensity obtained was 4201.25 W/$m^{2},$ and the acoustical power can be calculated by Equation (6):

(6) W=I∗S

where W is the acoustical power, and S is the area perpendicular to the direction of propagation. Finally, we could calculate the acoustical power as about 0.105 W.

**Figure 6:**
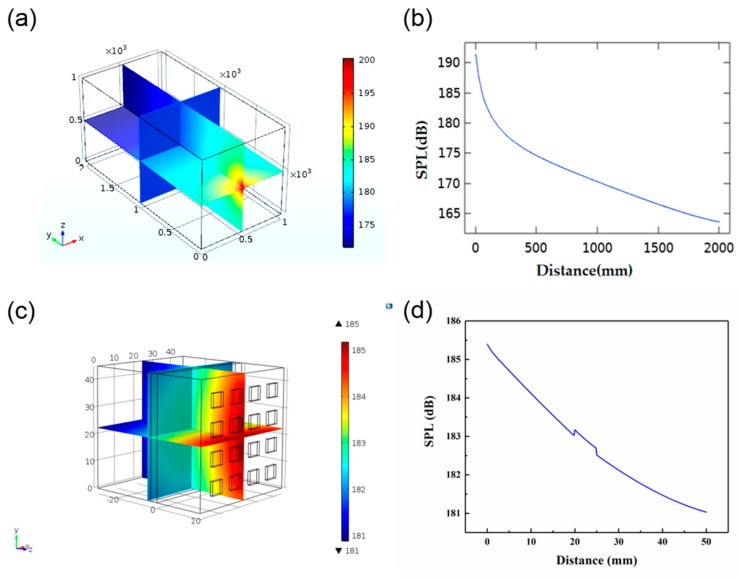
(**a**) Simulation of the distribution of sound field in water. (**b**) The relationship between sound pressure level (SPL) and distance. (**c**) Simulation model of the sound field through the skin tissue. (**d**) Changes in SPL through the skin tissue.

In order to study the acoustic energy distribution of the device in water, acoustic methods to simulate the sound field in water were used. A water tank model with a size of 1 m × 1 m × 2 m was set up to simulate the environment for actual testing in the future. We treated each piezoelectric element as a point source, and the value of each point source was 0.105 W, as calculated by Equation (6). Figure 6a shows a subdivision of the sound field obtained from the simulation. Figure 6b corresponds to the value of the SPL in the Y direction with the center of the array as the origin. As can be seen from the diagram, the SPL attenuated as the distance increased from 0 to 2000 mm. With the distance increasing, the attenuation gradually diminished from 191.35 to 163.57 dB. We also did a simulation of ultrasound penetration through the skin tissue. The size of the model was 45 mm × 45 mm × 50 mm, and the tissue width was 3 mm. As can be seen in Figure 6d, the SPL decreased as the distance increased. Due to the differences in acoustic parameters, such as density and sound velocity, the value of SPL changed dramatically at the point of contact between different substances.

#### 3.1.4. Ultrasonic Penetration of the Human Tissue

**Figure 7:**
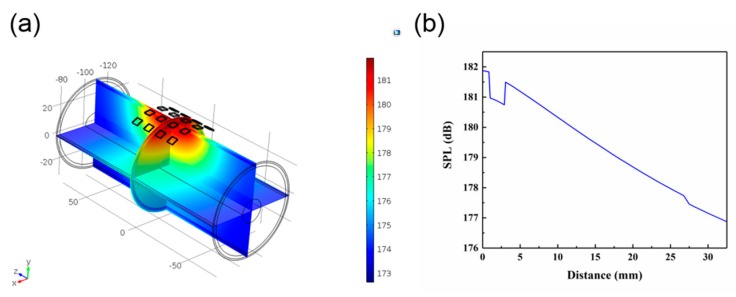
(**a**) Simulation of the distribution through the human tissue. (**b**) Changes in SPL through the human tissue.

Figure 7a show the simulation of ultrasonic penetration through the human tissue. According to anatomical characteristics, the upper arm is equivalent to a four-layer concentric cylinder composed of bone, muscle, fat, and skin. The thickness of each part is shown in Table 2. The length of the established cylinder was 150 mm, and the 4 × 4 PZT array was attached to the surface of the skin tissue. In order to simplify the simulation model, we omitted the PDMS layer. Figure 7b shows the change in SPL with increasing distance. As the ultrasound reached the bone, the SPL attenuated from 185.6 to 184.3 dB. When the PZT array was attached to the curved surface, it still worked effectively. However, there was a mutation with the increase of distance in the different medium interface (Figure 7b), which might be attributed to the different density of the medium and different sound speed in the different medium interface.

### 3.2. Experimental Results

#### 3.2.1. Measurement of the Resonant Frequency

**Figure 8:**
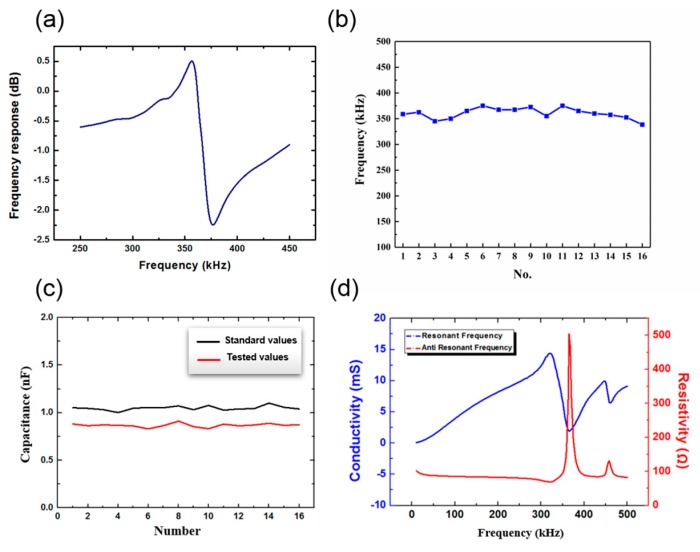
(**a**) The resonant frequency of the PZT. (**b**) The resonant frequency of all the PZTs in the array. (**c**) Capacitance test of the piezoelectric ceramic array. (**d**) The resonant frequency and the anti resonant frequency of the device in water.

The resonant frequency of the PZT elements in the array was measured and is shown in Figure 8a,b. It can be seen that the resonant frequency of PZT elements in the array was approximately 355 KHz within 20 KH, which is similar to the simulation results shown in Figure 5. In Figure 8c, both standard capacitive values and tested values after bonding of each PZT in the array are presented. The tested value was generally less than the standard, but the deviation was very small at only 0.181 nF. This indicates that the PZT units were fully bonded with the flexible electrode, and the device could conduct electricity normally. Figure 8d shows the resonant frequency and the anti resonant frequency of the device tested in water. The resonant frequency measured by an impedance analyzer was 321.15 KHz, and it was different from the data measured in air.

#### 3.2.2. Flexibility Test of the Device 

**Figure 9:**
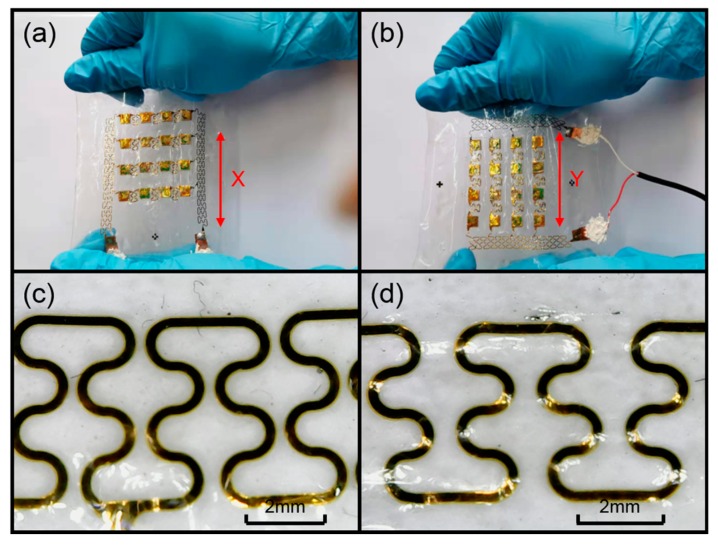
(**a**) Stretching the device along the x axis. (**b**) Stretching the device along the y axis. (**c**) The electrode in its initial state. (**d**) The electrode in its stretched state.

In order to verify that the device has excellent flexibility and can fit the skin surface, we carried out a tensile test. As shown in Figure 9a,b, we stretched the device along the x and y axes. We used a handheld microscope to take pictures of the serpentine electrode in its initial and stretched states. Figure 9c shows the diagram of the electrode in its initial state, and Figure 9d shows the stretched electrode. Comparing the two electrode diagrams, it can be seen that the internal structure of the electrode was not damaged when the device was stretched by 25%, which is greater than the maximum stretch of the skin.

#### 3.2.3. Ultrasonic Emission Experiment in Water

**Figure 10:**
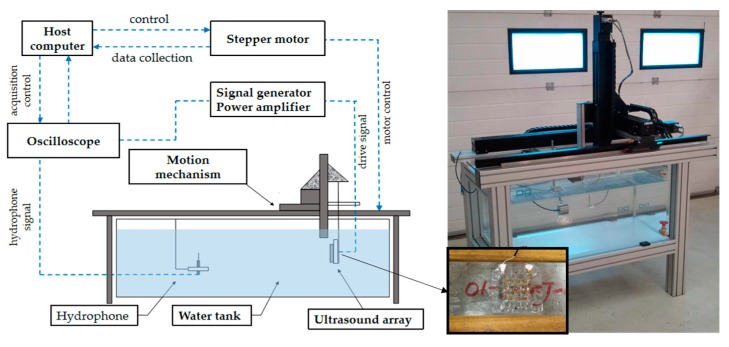
The schematic diagram of the test.

**Figure 11:**
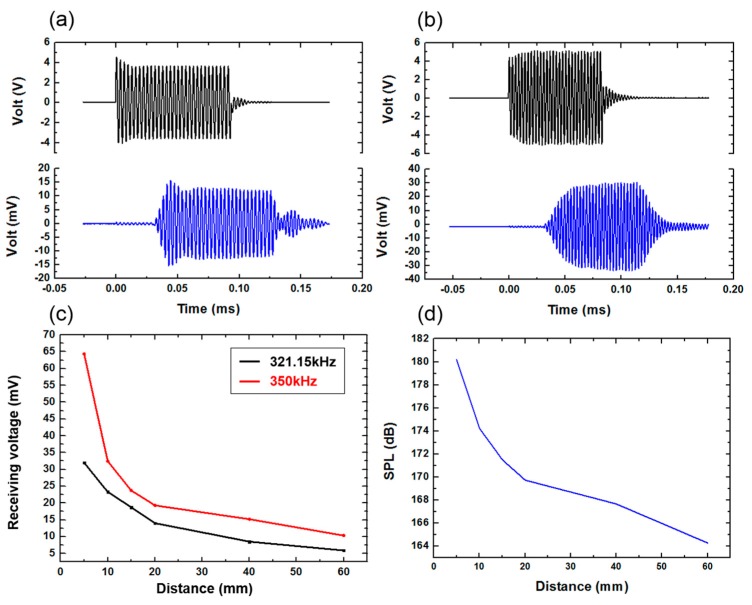
(**a**) The receiving signal at the frequency of 321.15 KHz. (**b**) The receiving signal at the frequency of 350 KHz. (**c**) Signal comparison at the frequency of 321.15 and 350 KHz. (**d**) The SPL tested at different distances.

A schematic diagram of the ultrasonic emission experiment is shown in Figure 10. Firstly, an antijamming cable was connected to the electrode of the device, and the connecting component was protected for waterproof treatment. Next, the device was fixed on a flat plate to make it convenient to carry out quantitative experimental analysis in the water tank. The other end of the cable was connected to the signal generator and the power amplifier. The hydrophone (TC4035, Teledyne RESON) was connected to the oscilloscope to accept the signal, and it was fixed in the water tank. The signal generator produced a sinusoidal signal with a voltage of 10 V, and the frequency was 321.15 KHz (measured by impedance analyzer), which was driven by a power amplifier. Figure 11a shows the results when the distance between the device and the hydrophone was 5 cm. It can be seen that the hydrophone received the signals from the FPMUT. The black line indicates the emission wave, and the blue line indicates the received wave.

With a constant change of excitation frequency, the maximum value of the received ultrasonic signal was found to be 350 KHz, as shown in Figure 11b. The strength of the ultrasound signal, which changed with the distance between the device and the hydrophone at the two different frequencies, can be seen in Figure 11c. A sinusoidal excitation signal with a frequency of 350 KHz allowed the ultrasonic power generated by the flexible piezoelectric ultrasonic transducer to be larger. Therefore, the frequency of 350 KHz was used in the following tests. When the distance between the device and the hydrophone was changed, the hydrophone was used to record the intensity of the signal received. The sound pressure level measured by the hydrophone can be obtained by the following equation:

(7) $$20\log\left(\frac{V_{p}}{\sqrt{2}}\right)-A$$

where $V_{p}$ is the measured peak-to-peak value of signal voltage, A is the sensitivity standard value of hydrophone. The standard sensitivity of this type of hydrophone is 213.057 dB. The SPL tested at different distances is shown in Figure 11d.

**Figure 12:**
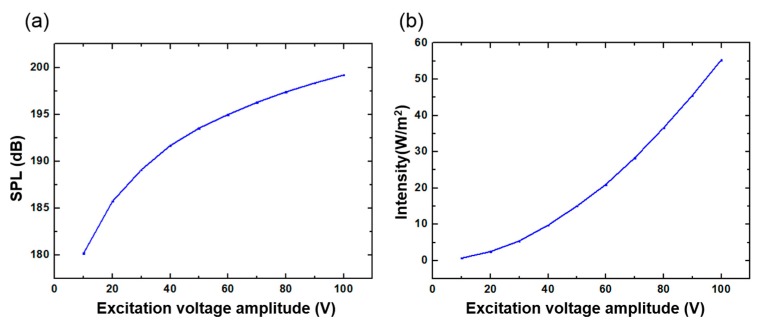
(**a**) The relationship between sound pressure level and excitation voltage amplitude. (**b**) The relationship between sound intensity and excitation voltage amplitude.

The SPL was also tested at different voltage amplitudes. As shown in Figure 12a, the sinusoidal excitation signal frequency was 350 KHz when the distance between the device and the hydrophone was limited to 5 cm. In order to more intuitively demonstrate the sound intensity of the ultrasonic waves generated by the FPMUT, the measured sound pressure level data was converted into sound intensity using Equations (8) and (9), as shown in Figure 12b. The two figures show that, when the frequency of the sinusoidal excitation signal was 350 KHz and the amplitude reached 100 V, the signal measured by the hydrophone at a distance of 5 cm from the flexible piezoelectric ultrasonic transducer had a sound intensity of 5.533 mW/cm^2^. Various studies have indicated that LIPU with frequency less than 10 MHz and intensity less than 30 mW/cm^2^ can shorten the healing period of bone injury. Because the minimum distance controlled by this experiment was 5 cm, which is bigger than the thickness of human tissue, the sound intensity measured from the tests was small. However, it can be seen from the current experimental data that the low-intensity ultrasound generated by the flexible piezoelectric ultrasonic transducer has the potential to assist in the treatment of fracture healing, and its safety performance depends on the magnitude of the excitation voltage and the length of the irradiation time.

(8) $$SPL=20\log\left(\frac{P_{e}}{P_{ref}}\right)$$

(9) $$I=\frac{P_{e}^{2}}{\rho c}$$

#### 3.2.4. Tissue Penetration Experiment

**Figure 13:**
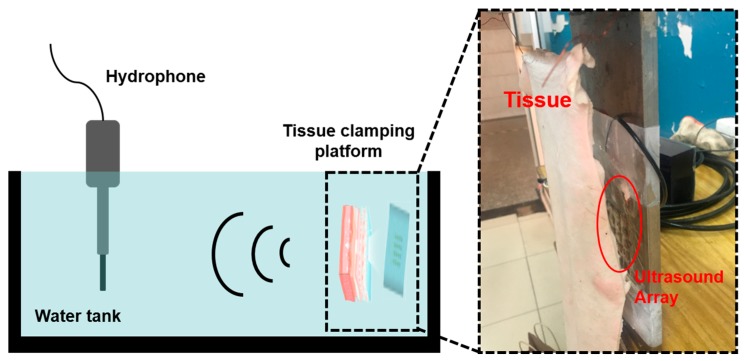
The test device of the penetration performance of ultrasound.

Ultrasound transmission to the skeletal tissue requires penetration through the skin, fat, and muscle tissues. The detailed tissue thickness of a human upper arm is given in Table 2. To help heal bone injury, the ultrasound must penetrate through 25.75 mm of tissue. In order to test the penetration performance of the ultrasound that was produced by the FPMUT, the distance between the device and the hydrophone was limited to 5 cm. The test device is shown in Figure 13. Different pork tissues were placed between the FPMUT, and the corresponding changes in the received ultrasonic signals are shown in Table 3. The data measured in the experiment were smaller than the data obtained in the simulation. On the one hand, the water in the water tank in the experiment was not pure water, and there were many impurities that would weaken the propagation of ultrasound. On the other hand, the test environment was far more complicated than the simulation. In the simulation, only the ideal situation was considered.

#### 3.2.5. Comparison with Some Previous Works

We compared our device with some previous works, and the results are given in Table 4. Our device operates mainly at low frequencies compared with other devices, and its tensile property is much better than those of other devices. In terms of application, it is mainly used as an adjuvant treatment for bone injury, which is very different from other processes. The proposed device is a great development in the application of flexible electronic devices with piezoelectric ultrasonic energy exchange. 

## 4. Conclusions

In this work, a FPMUT array was developed. With the combination of flexible interconnection, a flexible substrate, and piezoelectric ceramics, the device is flexible for good attachment to the skin. The measured resonant frequency of the single element was about 356.6 KHz. Ultrasonic emission experiments in water was carried out to study the ultrasonic output of the FPMUT. The ultrasonic wave produced by this device worked well for the penetration performance of different pork tissues. All of these results demonstrate that this device has great potential for medical applications, such as the adjuvant treatment of bone injury. We will do more experiments and research on it in the future.

## Figures and Tables

**Table 1:** Parameters used in simulation.

Material	Young’s Modulus	Poisson’s Ratio
PI	3.1 GPa	0.37
**Density (kg/m^3^)**	**Thickness (μm)**	**Displacement (mm)**
1300	4.8	2

**Table 2:** Density and thickness of different tissues.

Tissue	Density (g/cm^3^)	Thickness (mm)
Skin	1.109	0.75
Fat	0.911	3
Muscle	1.09	22
Bone	0.44	8

**Table 3:** Ultrasonic signals measured under different pork tissues.

Organization	Thickness (cm)	SPL (dB)
Pig skin	0.3	179.532
Fatty pork	3	177.376
Pork fat	1	179.819
Lean pork	0.5	179.415

**Table 4:** Comparison of this work with previous works.

Devices	Resonant Frequency	Tensile Ratio	Application
Mastronardi et al. [29]	F_0-1_ = 587.81 KHz F_0–2_ = 1.038 MHz F_0–3_ = 1.413 MHz	Not mentioned	Endoscopic analysis
Lee et al. [30]	F_700 μm_ = 694.4 KHz F_800 μm_ = 565.4 KHz F_900 μm_ = 430.8 KHz F_1200 μm_ = 289.3 KHz	Only bending deformation, little stretchability	Deep brain stimulation
Yang et al. [31]	2.016 MHz	9%	Ultrasound imaging
Wang et al. [32]	2 MHz	22.5%	Heart imaging
This work	350 KHz	25%	Healing of bone injury
